# Differential regulation of alanine aminotransferase homologues by abiotic stresses in wheat (*Triticum aestivum* L.) seedlings

**DOI:** 10.1007/s00299-012-1231-2

**Published:** 2012-02-12

**Authors:** Maria Kendziorek, Andrzej Paszkowski, Barbara Zagdańska

**Affiliations:** Biochemistry Department, Warsaw University of Life Sciences, Nowoursynowska 159, 02-776 Warsaw, Poland

**Keywords:** Wheat seedlings, Alanine aminotransferase, Glutamate: glyoxylate aminotransferase, Hypoxia, Nitrogen deficiency, Light

## Abstract

Wheat (*Triticum aestivum* L.) seedlings contain four alanine aminotransferase (AlaAT) homologues. Two of them encode AlaAT enzymes, whereas two homologues act as glumate:glyoxylate aminotransferase (GGAT). To address the function of the distinct AlaAT homologues a comparative examination of the changes in transcript level together with the enzyme activity and alanine and glutamate content in wheat seedlings subjected to low oxygen availability, nitrogen and light deficiency has been studied. Shoots of wheat seedlings were more tolerant to hypoxia than the roots as judging on the basis of enzyme activity and transcript level. Hypoxia induced AlaAT1 earlier in roots than in shoots, while AlaAT2 and GGAT were unaffected. The increase in AlaAT activity lagged behind the increase in alanine content. Nitrogen deficiency has little effect on the activity of GGAT. In contrast, lower activity of AlaAT and the level of mRNA for AlaAT1 and AlaAT2 in wheat seedlings growing on a nitrogen-free medium seems to indicate that AlaAT is regulated by the availability of nitrogen. Both AlaAT and GGAT activities were present in etiolated wheat seedlings but their activity was half of that observed in light-grown seedlings. Exposure of etiolated seedlings to light caused an increase in enzyme activities and up-regulated GGAT1. It is proposed that hypoxia-induced AlaAT1 and light-induced peroxisomal GGAT1 appears to be crucial for the regulation of energy availability in plants grown under unfavourable environmental conditions.

*Key message* In young wheat seedlings, both AlaAT and GGAT are down-regulated by nitrogen deficiency, whereas AlaAT1 is upregulated by hypoxia and GGAT1 by light.

## Introduction

Alanine aminotransferase (AlaAT) catalyses the reversible reaction of conversion of alanine and 2–oxoglutarate into pyruvate and glutamate. This pyridoxal phosphate-dependent enzyme plays a key role in plant metabolism by linking primary carbon metabolism with the synthesis of amino acids. AlaAT is wide distributed enzyme and its activity was found in leaves, roots and flower organs in *Arabidopsis thaliana* (Igarashi et al. 2003), fruit (Rech and Crouzet 1974) and in the inner endosperm tissues of developing seeds of *Oryza sativa* (Kikuchi et al. 1999). *Arabidopsis thaliana* (Igarashi et al. 2003; Liepman and Olsen 2003; Miyashita et al. 2007), *Glycine max* (Rocha et al. 2010) and *Medicago truncatula* (Ricoult et al. 2006) contain four genes coding for AlaAT. Two of them, *AlaAT1* and *AlaAT2* encode AlaAT (EC 2.6.1.2), whereas two others, namely *AtGGAT1* and *AtGGAT2*, have glutamate:glyoxylate aminotransferase (GGAT; EC 2.6.1.4) activity (Igarashi et al. 2003; Liepman and Olsen 2003). Subcellular fractionation analysis has shown that the GGAT activity was mainly located within peroxisomes (Liepman and Olsen 2003). However, AlaAT localisation was not examined in detail but in silico predictions indicated that AlaAT1 is located in the cytosol and AlaAT2 is a mitochondrial enzyme (Liepman and Olsen 2003; Ricoult et al. 2006). Presence of the multiple AlaAT activities in different subcellular compartments appears to be directly associated with their metabolic role. In C_4_ plant *Panicum miliaceum* cytosolic AlaAT was found in both mesophyll and bundle sheath, therefore it has been suggested to be involved in transfer of pyruvate between these cell types (Son and Sugiyama 1992). Peroxisomal AlaAT exhibiting GGAT activity has been implicated in regulation of photorespiration (Fukao et al. 2002; Liepman and Olsen 2003; Igarashi et al. 2003). To confirm that GGAT1 is a peroxisomal enzyme involved in photorespiration, aoat1-1 mutants deficient in GGAT1 were isolated. These plants in normal light intensity and CO_2_ concentration exhibited all features typical to photorespiratory mutants (Igarashi et al. 2003). Moreover, transcript level for the gene encoding GGAT1 was undetectable and AlaAT and GGAT activities were significantly lower than in wild-type plants. Besides, AlaAT seems to regulate the respiratory oxygen consumption via the activation of the alternative oxidase in mitochondria by increased concentration of pyruvate (Gupta et al. 2009).

The regulation of AlaAT activity during plant response to various environmental stresses, both abiotic and biotic has been reported for several plant species. Oxygen deficiency, usually caused by flooding, is one of adverse environmental factors seriously disturbing germination and plant development leading to gradual necrosis of root tissues and formation of clusters of aerenchyma (Subbaiah and Sachs 2003). Under hypoxia oxidative phosphorylation is inhibited, while glycolysis and fermentative pathways are stimulated resulting in the production of ethanol, lactate, succinate and malate (Good and Muench 1993). Concomitantly, a significant increase in the concentration of two amino acids: l-alanine and γ-aminobutyric acid (GABA) has been observed in roots of oxygen deficient plants (Roberts et al. 1992; Reggiani et al. 2000). Alanine synthesis from pyruvate has at least two advantages: it enables to conserve carbon skeletons that otherwise would be lost by ethanolic fermentation pathway (Good and Muench 1992, 1993; Muench et al. 1994) and decreases the availability of pyruvate to prevent pyruvate accumulation and thus, reduces the consumption of oxygen (Gupta et al. 2009).

Due to important role in tolerance of hypoxia, AlaAT has been recently a subject of intense and detailed studies (de Sousa and Sodek 2003; Ricoult et al. 2006; Miyashita et al. 2007; Rocha et al. 2010). Hypoxia induced AlaAT and increased transcript levels for the genes encoding AlaATs has been reported in barley (*Hordeum vulgare*), maize (*Zea mays*) and soybean (*Glycine max*) roots (Good and Muench 1992, 1993; Muench et al. 1994; de Sousa and Sodek 2003) and *Medicago truncatula* (Ricoult et al. 2006).

The elevated activity of AlaAT seems to be equally important for plants recovering from hypoxia/anoxia. In roots of soybean AlaAT activity continued to increase during hypoxia despite that the accumulation of alanine had reached its maximum (de Sousa and Sodek 2003). Return to normoxia decreased the alanine level rapidly to pre-hypoxic level within 24 h. Therefore it was suggested that AlaAT is involved in degradation of alanine after low oxygen stress relief. This finding has been confirmed by Miyashita et al. (2007) who showed that the *Arabidopsis* AlaAT1 knock-out mutant (alaat1-1) was able to accumulate more alanine during hypoxia than wild type plants and the decline in accumulated alanine was delayed during recovery period. It appears that AlaAT plays limited role in alanine synthesis under low oxygen conditions, while elevated levels of AlaAT activity enables to convert rapidly the accumulated alanine back into pyruvate during recovery phase (Miyashita et al. 2007).

It should be underlined that the role of AlaAT is not limited to hypoxic conditions only. The induction of AlaAT and its expression is regulated by light and nitrogen deficiency in *Panicum miliaceum* (Son et al. 1992) and *Zea mays* recovered from N-deficiency (Muench et al. 1998). Recent studies carried out on transgenic *Brassica napus* (Good et al. 2007) and *Oryza sativa* (Shrawat et al. 2008) plants with introduced barley AlaAT gene were characterized by a much higher efficiency of nitrogen assimilation in comparison to unmodified (wild) plants.

Plants growing in natural environment experience changes in light quality and quantity, extremes of temperature, air humidity and soil water availability which significantly alter the plant growth and development and even may lead to plant death. Plants cannot escape from these environmental constraints and have developed different mechanisms to cope with detrimental effects of unfavourable conditions. The maintenance of functional integrity in plants facing adverse environmental conditions requires physiological and biochemical adjustments. Therefore, acclimation to different environmental conditions requires stress-induced reprogramming of gene expression resulting in the reorganization of plant metabolism and achievement of a new state of cellular homeostasis. However, such reorientation of metabolism requires an increased need for energy expenditure (Zagdańska 1995).

In the present work we have attempted to check whether AlaAT and GGAT are involved in regulation of energy availability in plants under unfavorable growth conditions such as hypoxia, nitrogen deprivation and light deficiency. The objects of our studies were wheat seedlings at early stage of growth because they may be considered as carbon heterotrophs. Their development depends exclusively on seed reserves (Bogdan and Zagdańska 2009) and neither light nor the exogenously supplied sugars had statistically significant effects on accumulation of dry matter (Miazek et al. 2001; Bogdan and Zagdańska 2006) since photosynthesis genes are expressed in the later phase of growth (Jang and Sheen 1994; Loza-Tavera et al. 1990).

## Materials and methods

### Plant growth conditions

Experiments were carried out on seedlings of wheat (*Triticum aestivum* L. cv Jasna). Seeds were surface-sterilised with 0.5% NaOCl solution for 30 min and then rinsed several times with distilled water. Seeds were transferred to plastic containers with wire gauze as mechanical support and hydroponic system supplied with continuously aerated Knop solution (3.5 mM Ca(NO_3_)_2,_ 1.5 mM KNO_3_, 1 mM KCl, 1 mM KH_2_PO_4_, 0.6 mM MgSO_4_ and 0.1 mM ferric citrate) supplemented with trace elements according to Hoagland and Arnon (1950). Seedlings were grown in a growth chamber at the day/night temperature of 23/18°C, relative humidity of 80% and irradiance of 130 μE m^−2 ^s^−1^ for 16 h. Part of 10-day-old seedlings was subjected to hypoxia conditions. Hypoxia was imposed by submerging roots and shoots up to 1/3 length in flasks containing Knop solution covered by about 3.5 cm layer of oil to prevent gas exchange. Seedlings subjected to nitrogen deprivation were transferred to the N-free Knop solution containing KCl and CaCl_2_ instead of KNO_3_ and Ca(NO_3_)_2_ to keep the potassium and calcium ions concentration at 3.5 mM. To examine the effect of darkness, seeds germinated in darkness in 23°C. Simultaneously seeds germinated for 6 days in the growth chamber (control). After 2 or 4 days of germination in darkness seedlings were transferred to the growth chamber.

### Aminotransferase activity assay

Plant material was ground with a mortar and pestle to a fine powder in liquid nitrogen. The ground samples (1 g) were extracted in five volumes of 50 mM Tris/HCl pH 7.5 containing 1 mM EDTA, 1 mM PMSF, 0.1 mM pyridoxal phosphate, 10 mM mercaptoethanol and 10% sorbitol and mixed in T25 Ultra-Turrax homogeniser. Samples were then clarified by centrifugation at 16,000*g* for 20 min.

The AlaAT (EC 2.6.1.2) enzyme activity was determined at 25°C in a continuous assay by coupling the reaction of lactate dehydrogenase (LDH) to NADH oxidation (Horder and Rej 1983). The reaction mixture contained 250 mM l-alanine, 15 mM 2-oxoglutarate, 20 μM pyridoxal phosphate, 0.13 mM NADH, 100 mM Tris/glycine pH 7.3 and three units of LDH. The reaction was started by adding the enzyme extract and formation of pyruvate was monitored at 340 nm. Specific activity of AlaAT was expressed in micromoles of the oxoacid formed per minute (U) and per milligrams of protein.

The GGAT (EC 2.6.1.4) enzyme activity was determined at 30°C in a discontinuous assay according to Rowsell et al. (1972). Incubation mixture in volume of 0.65 ml contained 15.4 mM l-glutamate, 5 mM glyoxylate, 20 μM PLP, 77 mM K-phosphate buffer pH 7.5 and the enzyme extract. The enzyme was pre-incubated with the amino acid substrate for 10 min at 30°C. The reaction was started by adding glyoxylate to the mixture and was stopped after 15 min by adding 0.1 ml of 10% trichloroacetic acid (TCA). The amount of 2-oxoglutarate formed in this reaction was determined spectrophotometrically at 340 nm using NADH and glutamate dehydrogenase (GDH) in the presence of ammonium ions. After termination of reaction, 0.2 M K-phosphate buffer pH 7.5 was added to the mixture to a volume of 2.8 ml. Subsequently, the mixture was supplemented with 0.1 ml 1.5 M NH_4_Cl, 0.1 ml 4.0 mM NADH and 5 U GDH.

Protein was determined according to Bradford (1976) with bovine serum albumin as a standard.

### Native PAGE and detection of enzymatic activity in gels

Polyacrylamide gel (12%) was prepared in 50 mM Tris/glycine buffer, pH 9.1 containing 10% glycerol and 10 mM l-alanine. Electrophoresis was run in 50 mM Tris/glycine pH 9.1 for 5 h. Gels were stained for the l-alanine:2-oxoglutarate aminotransferase activity according to Hatch and Mau (1972). They were first incubated for 30 min at 4°C in mixture containing 0.14 mM K-phosphate buffer pH 7.5 and 10 U/ml lactate dehydrogenase. The mixture was warmed to room temperature, supplemented with 34 mM l-alanine, 10 mM 2-oxoglutarate and 0.6 mM NADH and incubated for about 30 min. Then the gels were washed with deionised water and photographed under UV light. Concomitantly, glutamate and glyoxylate were used as substrates. However, activity bands with these substrates were not seen as clear bands and some smears appeared. Therefore, only gels staining with alanine and 2-oxoglutarate as substrates are presented.

### Determination of l-alanine and l-glutamate content

To determine changes in the concentration of l-alanine and l-glutamate in leaves and roots of wheat seedlings exposed to hypoxia, samples of 1 g fresh weight were ground in liquid nitrogen to a fine powder and then extracted with 5 ml of 20 mM K-phosphate buffer pH 7.5. Extracts were deproteinized with 6% TCA and then centrifuged for 20 min at 15,000*g*. Amino acids derivatives were separated by reverse-phase C8 column (Waters) attached to HPLC system (Waters) with a fluorescent detector and the sample tray with the function of auto-mixing of the contents of the vials. Pre-column fluorescent derivatives were made by mixing 150 μl of the extract with 450 μl of o-phthalaldehyde solution. From 50 to 100 μl of the mixture as supplied to a column depending on the initial content of proteins in the extract. As eluents, 60 mM phosphate buffer pH 6.8 (phase A) and 80% acetonitrile (phase B) were used. Amino acids were eluted from the column with a gradient of 0–80% acetonitrile. Detection of the fluorescent amino acid derivatives was carried out at 425 nm.

### RNA extraction and reverse transcription

Total RNA was isolated according to Chomczynski and Sacchi (1987). For RNA isolation, samples of shoot (100 g) or root (150 g) were used in each isolation. Genomic DNA was removed by DNase digestion (Sigma) according to manufacturer protocol. The quality of RNA was determined electrophoretically on 1% agarose gels containing formaldehyde and MOPS buffer and spectrophotometrically using GeneQuant™ 100 spectrophotometer (GE Healtcare). Absorbance was measured at 260 nm (for nucleic acids), 280 nm (for protein) and 230 nm (for sugars). The purity of RNA was calculated from the absorbance ratio A_260_/A_280_ and A_260_/A_230_. Isolated RNA was used to synthesise cDNA matrix for PCR and Real Time PCR reactions. The reversed trancription was carried out at 42°C for 1 h. RevertAid™ First Strand cDNA Synthesis Kit (Fermentas) containing Random Hexamer primers was used according to manufacturer protocol.

### PCR and seqence analysis

Polymerase Chain Reaction (PCR) was carried out in 2720 Thermal Cycler (Applied Biosystems). ReadyMix™ Taq RCR (Sigma) reagent kit was used according to manufacturer protocol. Specific primers used in reaction were desinged using IDT OligoAnalyzer 3.1 (http://eu.idtdna.com/analyzer/Applications/OligoAnalyzer/) and OligoCalc: Oligonucleotide Properties Calculator (http://www.basic.northwestern.edu/biotools/oligocalc.html) and synthesized in DNA Sequencing and Oligonucleotide Synthesis Laboratory, Oligo.pl, IBB PAN (Institute of Biochemistry and Biophysics Polish Academy of Sciences).

**Table:** 

Gene	Forward primer	Reverse primer
AlaAT1	CCGGAGAAGACGAGAAGATG	ACTGATTGCCGGGTACTGAC
AlaAT2	CATCGAGACCGAGTGAAATGA	CATTGCCTTGATCTTGTCCTC
GGAT1	AGGTTCTTGCGGAGGAAAAC	GAACCGACGACAATTCCAGT
GGAT2	GTATGCTGTGCGTGGAGAGA	TAACCACCAAAGCCCTAACG

Thrity cycles of the reaction consisting of matrix denaturation (at 94°C for 45 s), annealing (at 57°C for 30 s) and elongation (at 72°C for 60 s) was repeated to achieve final product.

PCR reaction product was cloned into pCR4-TOPO vector (Invitrogen) and competent One Shot TOP10 Chemically Competent *E. coli* (Invitrogen) were transformed as described in manufacturer’s protocol. Plasmids were extracted according to method described by Birnboim and Doly (1979) with slight modification. Obtained products were sequenced using ABI Prism Big-Dye Terminator Cycle Sequencing kit and DNA ABI Prism 3730 (Applied Biosystems) analyser in DNA Sequencing and Oligonucleotide Synthesis Laboratory, Oligo.pl, IBB PAN (Institute of Biochemistry and Biophysics Polish Academy of Sciences).

### Real-time PCR (RT-PCR)

Real Time PCR was performed in Light Cycler 2.0 (Roche) with the LC FastStart DNA Master SYBR Green I (Roche) according to manufacturer protocol. 0.15 mM concentration of primers and 3.0 mM or 2.5 mM for 18S rRNA MgCl_2_ were used. 8–9 μl of reagents mixture and 2 or 1 μl of cDNA matrix, respectively, was added to capillaries. Hybridization was carried out at 62°C for AlaATs and 18S rRNA or at 60°C for alcohol dehydrogenase 1 (ADH 1) and nitrogen reductase (NR). Results were analysed using LightCycler Software 4.1.

Primers used in reactions

**Table:** 

Gene	Forward primer	Reverse primer
AlaAT1	CCGGAGAAGACGAGAAGATG	CACTGTAAGCACCTAAACCG
AlaAT2	CATCGAGACCGAGTGAAATGA	CACCAACCAGGAGGAGATAG
GGAT1	AGGTTCTTGCGGAGGAAAAC	CTTCCATGTAGCCTCCTCGT
GGAT2	GTATGCTGTGCGTGGAGAGA	GCTCCTGTTGCTCTTCCA
ADH1	AGGACGCCGAATTCAAGAC	TGAACTTCTCCACCTCCAG
NR	TCCGGTGGTGGTAAGAAGATC	TCCGGTGGTGGTAAGAAGATC
18S rRNA	GATCCATTGGAGGGCAAGTC	GATGCTTGCTTTGAGCACTC

### Statistical analysis of data

The obtained results were subjected to statistical analysis using StatGraphics Centurion XVI and Microsoft Excel XP. Values presented in graphs are mean ± SD.

## Results

### Characterization of AlaAT and GGAT in wheat seedlings

**Fig. 1:**
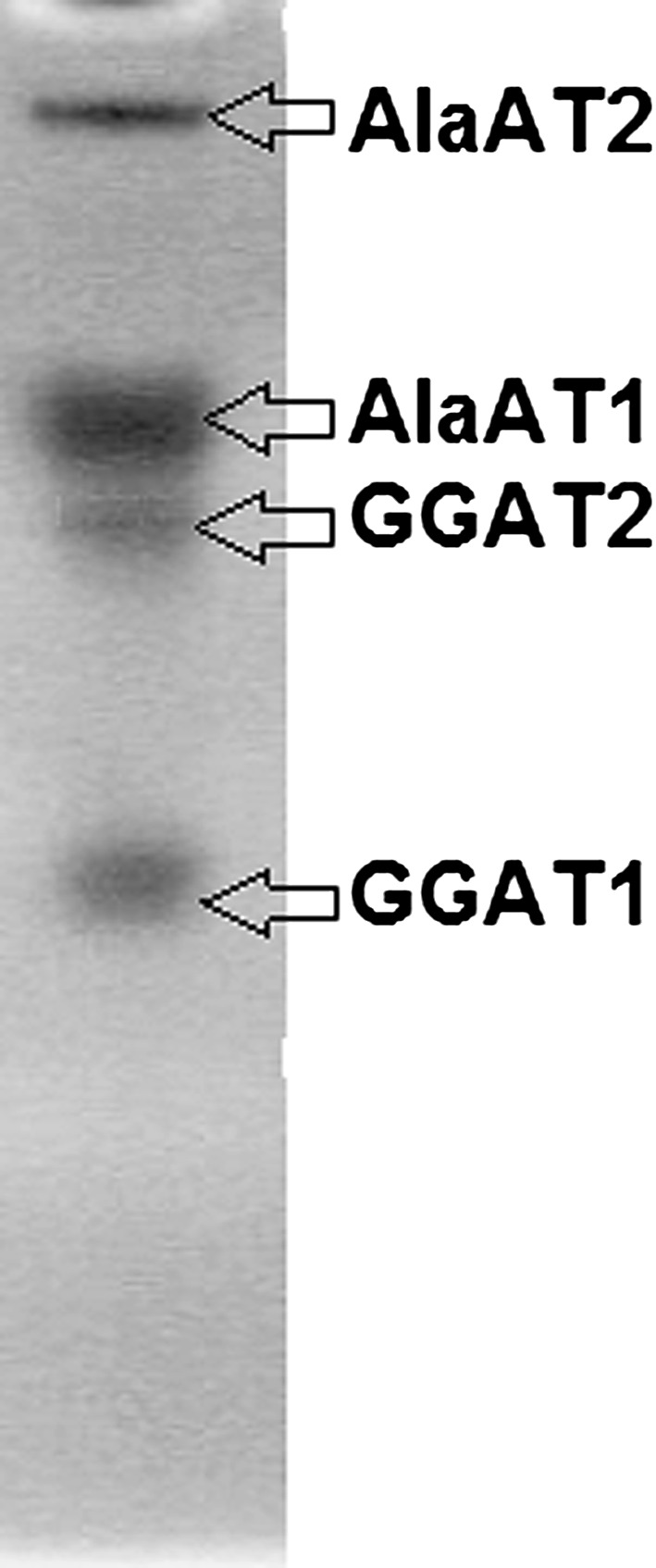
Native PAGE of alanine aminotransferase (AlaAT) homologues in shoots of wheat seedlings revealed by activity staining. An equal activity of AlaAT and GGAT (30 mU) was used for each lane

AlaAT activity gels showed that crude extracts of shoots of wheat seedlings contain four AlaAT homologues (Fig. 1). They were identified on a basis of native gel electrophoresis of AlaAT homologues’ preparations, purified and separated on anion exchange Protein Pak Q 8HR column attached to HPLC (Waters) system (data not shown). A cytosol form of the enzyme (AlaAT1) seems to be the predominant AlaAT homologue detected whereas a mitochondrial form of the enzyme (AlaAT2) is low in abundance. Similarly, GGAT1 seems to be the more active than the GGAT2 form.

**Fig. 2:**
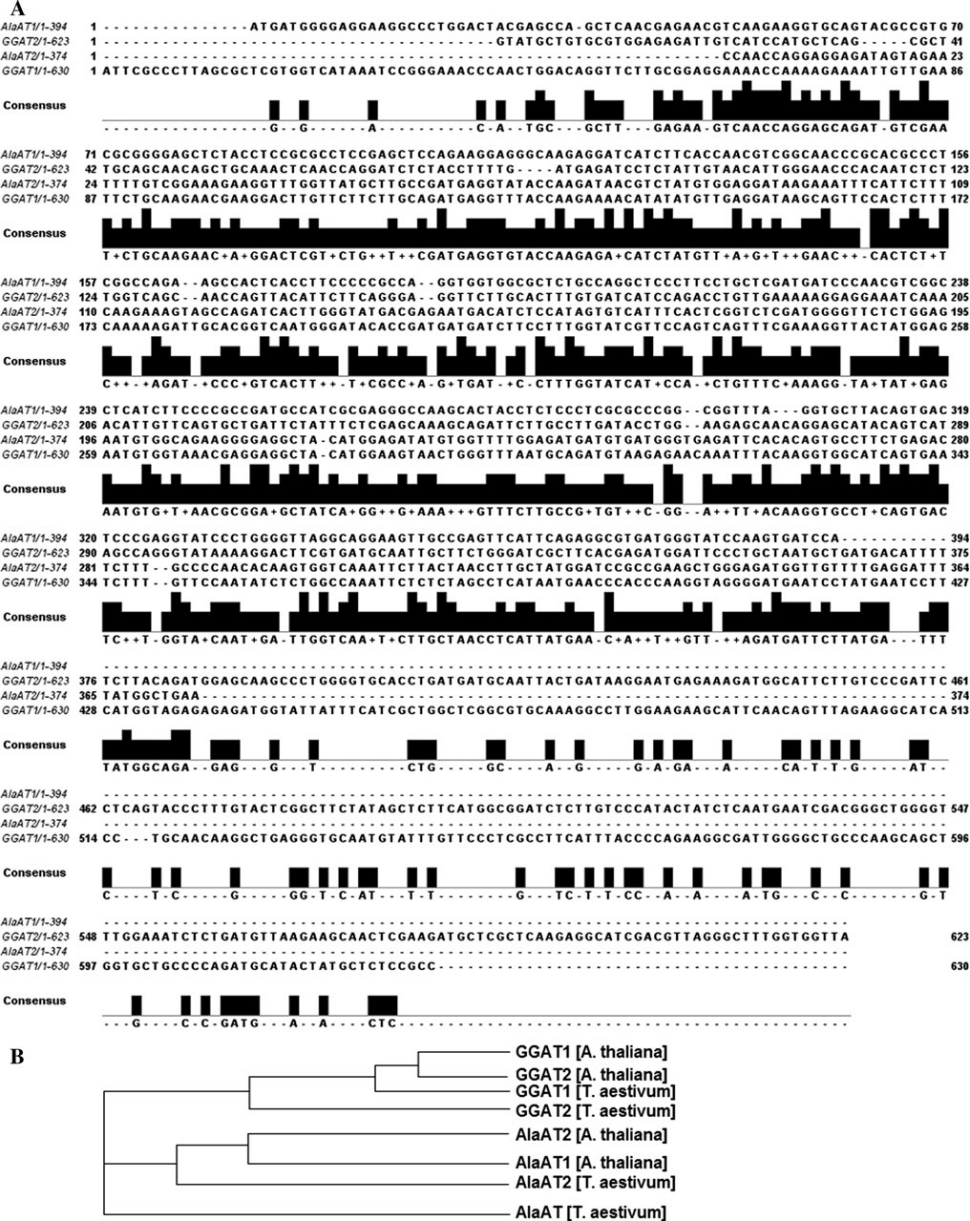
Sequence analysis of AlaAT homologues from wheat. **a** Alignment of obtained sequences by ClustalX 1.83.** b** Phylogenetic tree of the AlaAT enzyme family. Comparison was performed using coding sequences of wheat and all fully sequenced for AlaAT genes in *Arabidopsis* by Clustal W2

From the wheat genome sequencing information available in the public data domain (TIGR, http://compbio.dfci.harvard.edu/cgi-bin/tgi/gimain.pl?gudb=wheat) it was searched for the the presence of AlaAT isoenzymes using the tBLASTn. Four fragments of cDNA encoding wheat AlaATs corresponding to *Arabidopsis* genes (At1g72330, At1g17290, At1g23310, At1g70580) were obtained (Fig. 2a). A phylogenetic analysis of AlaAT from *Arabidopsis* *thaliana* and *Triticum aestivum* performed using ClustalW programme (Higgins et al. 1994) showed that two of them were closely related to Arabidopsis AlaAT1 and AlaAT2 while other two appeared to be related to GGAT1 and GGAT2 (Fig. 2b).

### Effect of hypoxia on AlaAT and GGAT

**Fig. 3:**
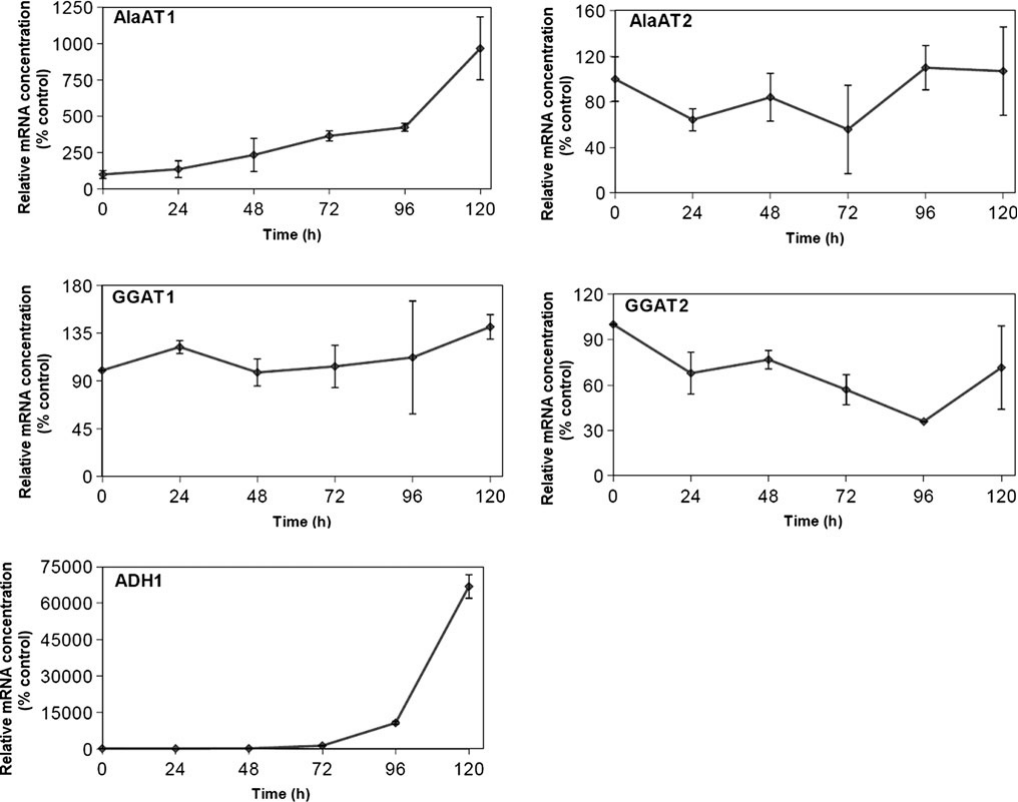
Transcript level for the genes encoding AlaAT homologues in shoots of wheat seedlings subjected to hypoxia. Quantitive Real Time PCR was performed on cDNA synthesized on the basis of total RNA extracted from shoots of control seedlings i.e. grown under optimal conditions and harvested 24, 48, 72, 96 and 120 h after induction of hypoxia. Development of hypoxia has been monitored by quantification of the response of gene encoding alcohol dehydrogenase (ADH1). Relative mRNA concentration is expressed in arbitrary units corresponding to the ratio of cDNA copy number of studied gene/18S rRNAcDNA copy number and presented as % of the relative mRNA concentration in control seedlings i.e. grown under optimal conditions. Results are the mean of three replicates ± SD

**Fig. 4:**
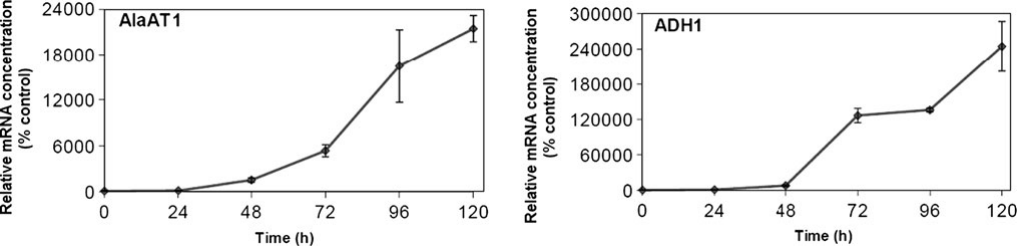
Transcript level for the genes encoding AlaAT homologues in roots of wheat subjected to hypoxia. Quantitive Real-Time PCR was performed on cDNA synthesized on the basis of total RNA extracted from roots of control plants and plants harvested 24, 48, 72, 96 and 120 h after induction of hypoxia. Other details are as described in Fig. 3. Results are the mean of three replicates ± SD

Expression profiles of AlaATs were studied by real-time quantitative PCR in shoots and roots of wheat seedlings subjected to hypoxia. The gene encoding the 18S rRNA has been used as a constitutive control. The obtained results referred to relative mRNA concentration were expressed in arbitrary unit (AU) corresponding to the ratio of cDNA copy number of studied gene/18S rRNA cDNA copy number. Changes in transcript levels are presented as percentage of the relative mRNA concentration in control plants. Development of hypoxic metabolism in young wheat seedlings has been monitored by quantification of the response of gene encoding the fermentative enzyme, alcohol dehydrogenase 1 (ADH1). In response to oxygen deficiency, the concentration of mRNA for ADH1 started to increase in shoots of wheat seedlings after 72 h of hypoxic treatment (Fig. 3), whereas in roots of wheat seedlings after 48 h of hypoxic treatment (Fig. 4).

Hypoxia-induced increase in transcript levels was observed only for AlaAT1 gene (Figs. 3, 4). AlaAT1 mRNA concentration was about tenfold higher in shoots of seedlings subjected to 120 h hypoxia treatment (Fig. 3), whereas in roots its concentration increased about 200-fold as compared to untreated control seedlings. The expressions of other three genes encoding wheat AlaATs appeared to be unaffected by hypoxia. These results indicate that AlaAT1 is the major gene expressed in shoots and roots of wheat seedlings among the four members of the gene family.

**Fig. 5:**
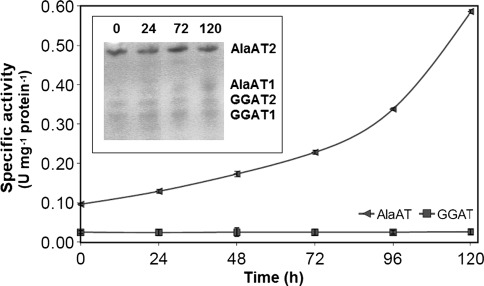
AlaAT and GGAT specific activities in shoots of control wheat seedlings and seedlings harvested 24, 48, 72 and 120 h after induction of hypoxia. Changes in activity of individual AlaAT and GGAT revealed by activity staining are given in inset graph. Results are the mean of three replicates ± SD

**Fig. 6:**
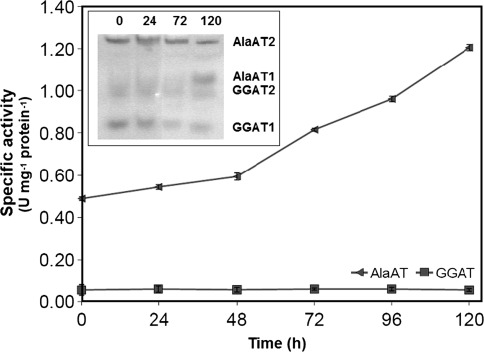
AlaAT and GGAT specific activities in roots of control wheat seedlings and seedlings harvested 24, 48, 72 and 120 h after induction of hypoxia. Changes in activity of individual AlaAT and GGAT revealed by activity staining are given in inset graph. Results are the mean of three replicates ± SD

The change in specific activity of AlaAT, as induced by hypoxia, was very similar between shoots and roots of wheat seedlings (Figs. 5, 6). Specific activity of AlaAT increased steadily upon hypoxia while GGAT activity remained practically unchanged. A hypoxia-induced increase in AlaAT specific activity was visualized by a higher intensity of the AlaAT1 activity band in both shoot (Fig. 5) and root (Fig. 6) extracts. As judged by the native gel assay, no changes in intensity of other three bands of AlaAT activity were observed.

**Fig. 7:**
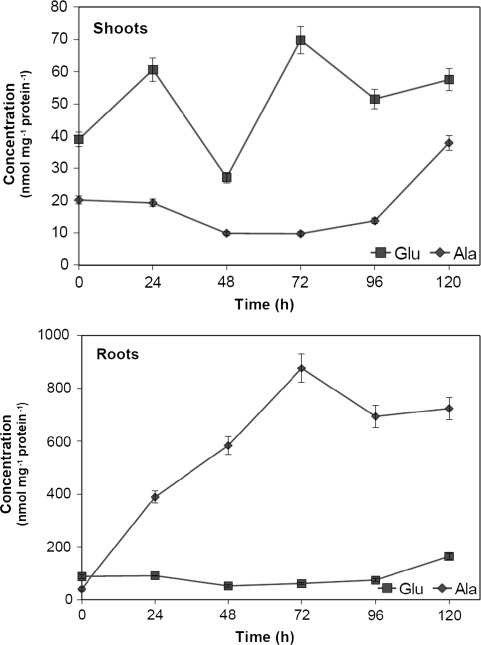
Alanine and glutamate concentration quantified by RP-HPLC in shoots and roots of wheat seedlings subjected to hypoxia. Amino acids were extracted from shoots and roots of control seedlings and seedlings harvested 24, 48, 72, 96 and 120 h after induction of hypoxia. Results are the mean of three replicates ± SD

To analyze the involvement of AlaAT in alanine accumulation during oxygen deficiency, the changes in alanine and glutamate contents were investigated. Alanine concentration in seedling shoots decreased by about 50% after 48 h of hypoxia and then started to increase and was about twofold higher after 120 h of hypoxia as compared to control seedlings (Fig. 7). Glutamate concentration remained practically unchanged in seedling roots upon hypoxia but it varied considerably in seedling shoots and increased 1.5-fold after 120 h of hypoxia. As a result, alanine accumulated rapidly in roots under hypoxia. After 24 h of hypoxia treatment alanine content was about tenfold higher and continued to increase reaching maximum after 72 h hypoxia and then started to decrease. Glutamate concentration, however, decreased by about 42% after 48 h hypoxia treatment and started to reach the final concentration twice as high as in control plants.

The ratio of glutamate to alanine content varied considerably upon hypoxia. In shoots and roots of control seedlings glutamate concentration was about twofold higher than alanine concentration. However, the Glu/Ala concentration ratio in shoots and roots of wheat subjected to hypoxia turned out to be opposite to each other. In shoots the ratio increased to reach maximum after 72 h of hypoxia and then decreased. In roots the Glu/Ala concentration ratio decreased and after 72 h of hypoxia the glutamate content was almost 14-fold lower than alanine content. During next 48 h of hypoxia the Glu/Ala ratio in roots increased about threefold but remained about ten times lower than in untreated control seedlings.

### AlaAT and GGAT activities upon nitrogen deprivation

**Fig. 8:**
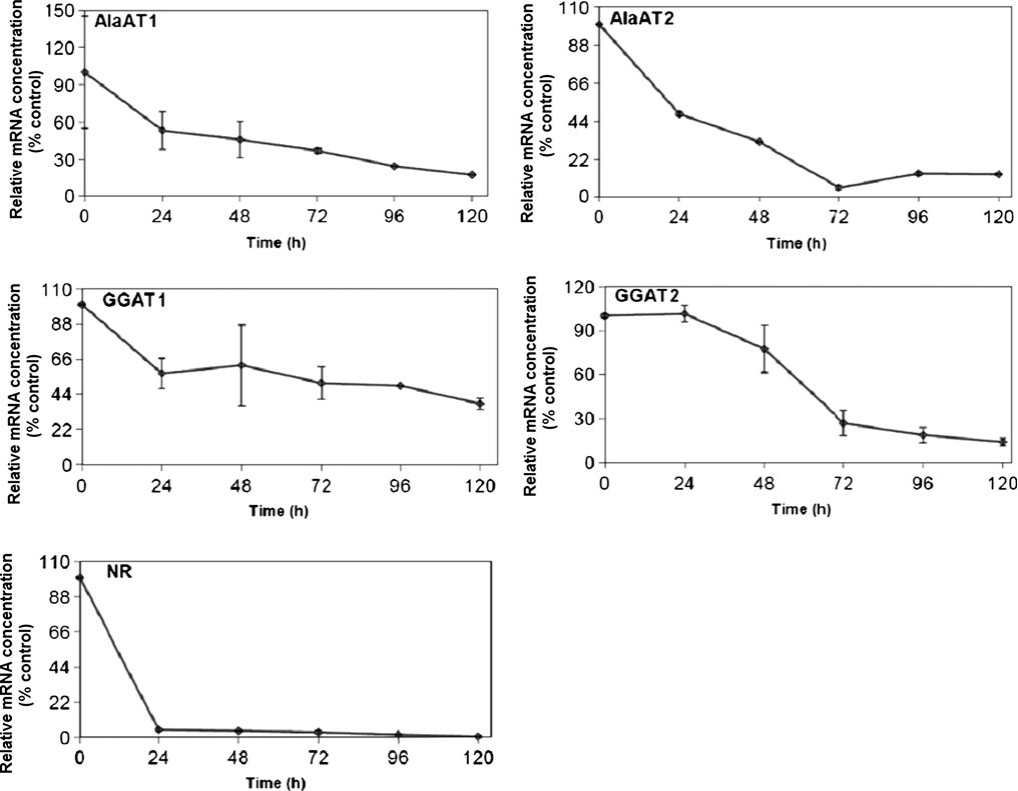
Transcript level for the genes encoding AlaAT homologues in shoots of wheat subjected to nitrogen deprivation. Quantitive Real Time PCR was performed on cDNA synthesized on the basis of total RNA extracted from shoots of control seedlings and seedlings harvested 24, 48, 72, 96 and 120 h after transfer to a nitrogen-free medium. Transcription level of nitrate reductase (NR) as a marker gene for nitrogen starvation was investigated. Results are the mean of three replicates ± SD

**Fig. 9:**
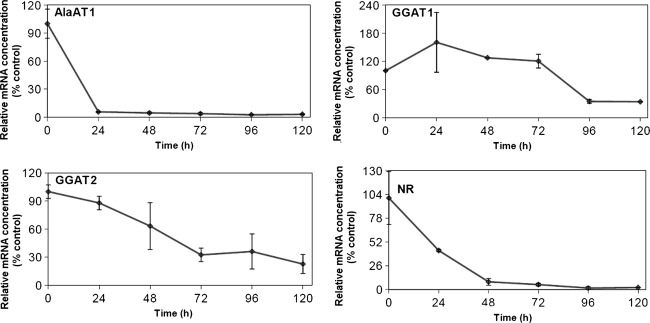
Transcript level for the genes encoding AlaAT and GGAT in roots of wheat subjected to nitrogen deprivation. Quantitive Real Time PCR was performed on cDNA synthesized on the basis of total RNA extracted from roots of control plants and plants harvested 24, 48, 72, 96 and 120 h after transfer to a nitrogen-free medium. Results are the mean of three replicates ± SD

To investigate the impact of nitrogen deprivation on AlaAT, the changes in transcript levels, enzyme specific activity and a quantitative gel assays were studied in shoots and roots of wheat seedlings grown in the absence of nitrates after 14 days of growth in the presence of nitrates in Knop solution. Transcription level of nitrate reductase (NR) as a marker gene for nitrogen starvation was investigated (Figs. 8, 9). In response to N-deficiency, a rapid decrease in concentration of mRNA for NR was observed in both roots and shoots of wheat seedlings, the effect being more pronounced for wheat shoots.

After 24 h of wheat seedlings growth on a N-free medium mRNA concentration for all genes encoding AlaAT homologues decreased (Figs. 8, 9), the effect being more pronounced for AlaAT1. Nitrogen deprivation for 5 days resulted in about sixfold decrease of transcript levels of the gene encoding AlaAT1 in shoots and 33-fold decrease in roots of wheat seedlings. Expression of gene encoding AlaAT2 in shoots decreased about eightfold. The response of GGAT1 and GGAT2 transcripts for N-deprivation was delayed and less pronounced as compared to AlaATs.

**Fig. 10:**
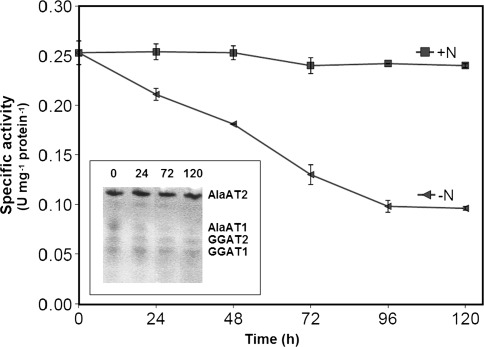
Effect of nitrogen deprivation on AlaAT specific activities in shoots of control seedlings (+N) and seedlings harvested 24, 48, 72, 96 and 120 h after transfer to a nitrogen-free medium (−N). Changes in activity of individual AlaAT and GGAT revealed by activity staining are given in inset graph. Results are the mean of three replicates ± SD

**Fig. 11:**
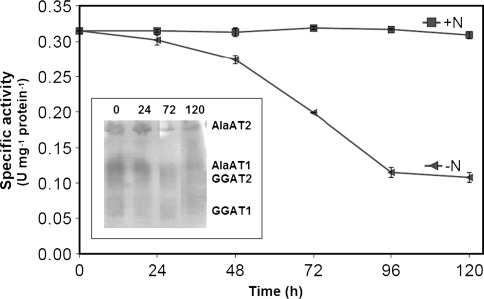
AlaAT specific activities in roots of control seedlings (+N) and seedlings harvested 24, 48, 72, 96 and 120 h after transfer to a nitrogen-free medium (−N). Changes in activity of individual AlaAT and GGAT revealed by activity staining are given in inset graph. Results are the mean of three replicates ± SD

As a result of 5-day growth of wheat seedlings on N-free medium significant decrease in specific activity of AlaAT both in shoots (Fig. 10) and roots (Fig. 11) was noted whereas the specific activity of GGAT remained unchanged (data not shown). AlaAT specific activity decreased steadily during 96 h nitrogen starvation and then it was stabilised. The native gel assay for AlaAT activity showed that the intensity of bands for AlaAT1 and AlaAT2 decreased only slightly. No changes in intensity of bands for GGAT1 and GGAT2 were noted.

### Light regulation of AlaAT and GGAT activity

**Fig. 12:**
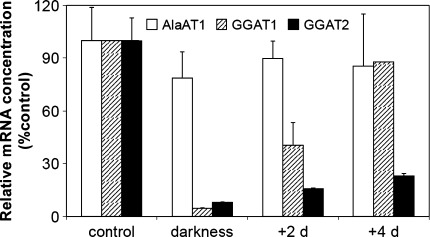
Light induction of transcription of the genes encoding AlaAT homologues. Quantitive Real Time PCR was performed on cDNA synthesized on the basis of total RNA extracted from shoots of etiolated seedlings and seedlings transferred for 2 or 4 days to light in comparison to control seedlings i.e. grown for 6 days in normal light conditions. Results are the mean of three replicates ± SD

To investigate light induction of AlaAT and GGAT in wheat seedlings, a comparison of transcript levels and enzyme activity in dark-grown seedlings and exposed to light was performed (Fig. 12). The highest mRNA concentration for genes encoding AlaAT homologues was observed in shoots of wheat seedlings growing for 6 days upon light (control seedlings). The transcription of genes encoding GGAT1 and GGAT2 was strongly depressed in darkness. Concentration of mRNA for GGAT1 and GGAT2 was lowest in shoots of etiolated seedlings, i.e. germinating in dark for 6 days, whereas the mRNA level for the gene encoding AlaAT1 was similar in dark- and light-grown seedlings. The inhibitory effect of darkness was reversible by light: in light-grown seedlings for 2 days the transcript levels for GGAT1 and GGAT2 were about nine- and twofold higher, respectively. Prolonged exposition of seedlings to light up to 4 days reversed almost completely the inhibitory effect of darkness in the case of GGAT1 transcripts.

**Fig. 13:**
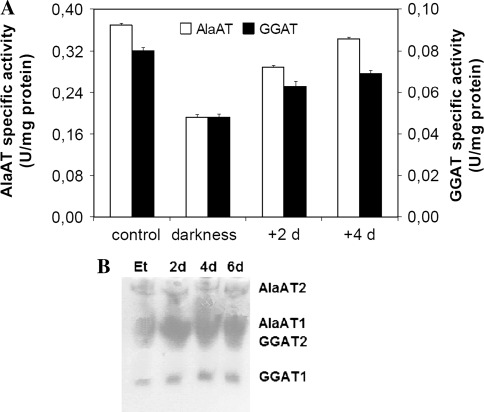
AlaAT and GGAT specific activities **a** in shoots of etiolated seedlings and seedlings transferred for 2 or 4 days to light in comparison to control seedlings i.e. grown for 6 days in normal light conditions. Results are the mean of three replicates ± SD. **b** Changes in activity of individual AlaAT and GGAT revealed by activity staining

Specific activities of both AlaAT and GGAT were lowest in shoots of etiolated seedlings and increased in plants exposed to light (Fig. 13a). The AlaAT and GGAT specific activity was about two times lower in etiolated seedlings in comparison to the light-grown seedlings. Exposure of seedlings to light increased activity of both AlaAT and GGAT almost twice. Intensity of bands for GGAT1 and GGAT2 increased in light-grown seedlings although intensity of GGAT2 band was slightly weaker in comparison to GGAT2 band (Fig. 13b).

## Discussion

The results presented here indicate that in wheat seedlings four alanine aminotransferase homologues are present. Based on the wheat sequencing data and comparison made between these sequences and homologues from *A. thaliana,* the existence of four genes in the wheat genome closely related to *Arabidopsis* alanine aminotransferase (AlaAT1 and Ala AT2) and glutamate: glyoxylate aminotransferase (GGAT1 and GGAT2) has been shown. Analysis of the expression profile of each member of the AlaAT gene family using RT-PCR revealed expression of these genes in both shoots and roots of young seedlings of wheat. This finding was supported by the activity staining of this enzyme on native PAGE. To the best of our knowledge, the present work is the first to report on the expression of AlaAT homologues in the young wheat seedlings.

Hypoxia tolerance referring to AlaAT activity was higher in shoots than in roots of wheat seedlings. Activity of AlaAT measured in vitro in extracts both from shoots and roots of wheat seedlings increased gradually up to 120 h of hypoxia. Activity staining on native PAGE revealed that the activity of AlaAT1 increased in plants subjected to oxygen deficiency, while other three homologues were not stimulated. In parallel with it, transcript level increased solely for the gene encoding AlaAT1 and the induction profile was similar to that of ADH1. However, hypoxia induced AlaAT1 earlier in roots (after 24 h of hypoxia treatment) than in shoots (after 48 h of hypoxia treatment). This is consistent with the same pattern of AlaAT induction in roots of maize (Muench et al. 1998), barley and rye (Good and Crosby 1989), soybean (de Sousa and Sodek 2003) and *Arabidopsis* (Miyashita et al. 2007). In contrast to gradual increase in AlaAT activity, the activity of GGAT was unaffected both in shoots and roots of wheat seedlings exposed to hypoxia. The observed divergent action of hypoxia on AlaAT and GGAT actvities illustrates that only enzymes unable to use glyoxylate as amino group acceptor may be involved in hypoxia tolerance.

Increased activity and transcript level of AlaAT was accompanied by a significant alanine accumulation in roots of plants subjected to low oxygen conditions. Alanine accumulated rapidly up to 72 h of hypoxia treatment while AlaAT specific activity and the transcription level for the gene encoding AlaAT1 still increased. This is consisitent with the finding that the increase in AlaAT activity lagged behind the increase in alanine content in roots of soybean (de Sousa and Sodek 2003). The observed differences in alanine and glutamate concentration between the shoots and roots of wheat subjected to hypoxia seem not to be the result of changes in the activity of AlaAT homologues with the exception of AlaAT1. Increased AlaAT1 transcript level, more than 20-fold higher in roots than in shoots indicates that the shoot response to hypoxia differs from that of roots. However, the results obtained draw attention to the role of alanine in hypoxia tolerance. Although alanine accumulation has been observed previously in roots under flooding (Limami et al. 2008), hypoxia and anoxia (de Sousa and Sodek 2003; Miyashita et al. 2007; Rocha et al. 2010), nitrogen deficit (Puiatti and Sodek 1999) and in shoots under water deficit (Drossopoulos et al. 1985) and low temperature (Mazzucotelli et al. 2006) the mechanism of alanine accumulation is still an intriquing aspect of plant response to unfovourable environmental conditions. The importance of alanine metabolism was discussed with respect to its ability to regulate the level of pyruvate under oxygen-depleted conditions (Rocha et al. 2010) and thus, control the rate of respiration and ATP production by activating the alternative oxidase (Millar et al. 1996; Vanlerberghe et al. 1999) or by converting pyruvate into ethanol or lactate. Additional advantage is that alanine as compatible solution can be up-regulated with little impact on cellular functions and thus, may stabilize the quaternary structure of proteins and membranes (Yancey 2005) and counteract the acidic effect of lactate (Ricoult et al. 2005).

Nitrogen deficiency decreased the specific activity of AlaAT and intensity of bands corresponding to AlaAT1 and AlaAT2 both in shoots and roots of wheat seedlings. On the contrary, neither decrease in the GGAT activity nor changes in the intensity of bands corresponding to GGAT1 and GGAT2 were noted, suggesting that nitrogen deficiency has little effect on the activity of these homologues. Although the level of mRNA for all four AlaAT homologues decreased in shoots of wheat seedlings grown on a nitrogen-free medium the largest decrease was observed for the AlaAT2 and AlaAT1 transcript levels. This is consistent with the observation that AlaAT is regulated by the availability of nitrogen to a greater extent than GGAT. The importance of AlaAT in nitrogen metabolism has been recently confirmed in experiments with oilseed rape (Good et al. 2007) and rice (Shrawat et al. 2008) with introduced AlaAT gene from barley.

Both AlaAT and GGAT activities were present in etiolated wheat seedlings and increased in plants exposed to light as it has been shown for cucumber (Noguchi and Fujiwara 1982). Furthermore all four bands of activities of AlaAT homologues were visible on gels after native electrophoresis of dark-grown seedlings. Increased intensity of GGAT1 and GGAT2 bands upon light exposure clearly indicates the involvement of GGAT in the greening process. Changes in GGAT1 and GGAT2 transcription level confirmed the results obtained for enzymatic activity. In fact, mRNA level for both GGAT1 and GGAT2 increased significantly, with the increases of GGAT1 transcription level about twofold higher than for GGAT2. Given the fact that the band corresponding to GGAT1 was also more intense than the band corresponding to GGAT2 one can assume that this homologue is involved in greening process of wheat seedlings.

Taken together, the significance of this work is demonstration that in the wheat genome there are four genes closely related to *Arabidopsis* alanine aminotransferase (AlaAT1 and Ala AT2) and glutamate:glyoxylate aminotransferase (GGAT1 and GGAT2). The present study of wheat seedlings shows that AlaAT and GGAT are crucially linked to the ability of plants to withstand adverse environmental conditions and revealed major changes in regulation of these enzyme activities related to oxygen, nitrogen and light availability. The involvement of cytosol AlaAT1 in hypoxia tolerance and peroxisomal GGAT1 in plant greening suggests that these homologues play the complex essential role in the regulation of energy availability in plants grown under unfavourable environmental conditions. The obtained results open new research perspectives, since little is known about the involvement of this enzyme in plant response to pathogen attack, which is one of the main factors limiting the productivity of plants. Moreover, the involvement of AlaAT and GGAT in tolerance of other unfavourable conditions such as extreme temperature (cold, frost, and heat) or drought remains to be elucidated.
